# Genome-Wide Identification and Expression Profiling of the α-Amylase (*AMY*) Gene Family in Potato

**DOI:** 10.3390/genes15060793

**Published:** 2024-06-17

**Authors:** Yudan Duan, Liping Jin

**Affiliations:** State Key Laboratory of Vegetable Biobreeding/Key Laboratory of Biology and Genetic Improvement of Tuber and Root Crops of Ministry of Agriculture and Rural Affairs/Institute of Vegetables and Flowers, Chinese Academy of Agricultural Sciences, Beijing 100081, China; 82101201103@caas.cn

**Keywords:** potato, α-amylase, *AMY* gene family, genome-wide identification

## Abstract

Starch degradation provides energy and signaling molecules for plant growth, development, defense, and stress response. α-amylase (*AMY*) is one of the most important enzymes in this process. Potato tubers are rich in starch, and the hydrolysis of starch into sugar negatively impacts the frying quality of potato. Despite its importance, the *AMY* gene family has not been fully explored in potatoes. Here, we performed a detailed analysis of the *StAMY* gene family to determine its role in potato. Twenty *StAMY* genes were identified across the potato genome and were divided into three subgroups. The promoters of *StAMY* genes contained an array of *cis*-acting elements involved in growth and development, phytohormone signaling, and stress and defense responses. *StAMY8*, *StAMY9*, *StAMY12*, and *StAMY20* were specifically expressed in mature tubers. Different *StAMY* gene family members tended to be upregulated in response to β-aminobutyric acid (BABA), *Phytophthora infestans* (*P. infestans*), benzothiadiazole (BTH), heat, salt, and drought stress. In addition, different *StAMY* gene family members tended to be responsive to abscisic acid (ABA), indole-3-acetic acid (IAA), gibberellic acid (GA3), and 6-benzylaminopurine (BAP) treatment. These results suggest that *StAMY* gene family members may be involved in starch and sugar metabolism, defense, stress response, and phytohormone signaling. The results of this study may be applicable to other starchy crops and lay a foundation for further research on the functions and regulatory mechanisms of *AMY* genes.

## 1. Introduction

Potatoes (*Solanum tuberosum* L.) are the world’s third most important food crop [1,2]. Potatoes are cultivated in 160 countries around the world and are critically important to food security [3]. The starch content of potato tubers is considered one of the most critical agronomic traits in potato breeding. It typically ranges from 10% to 25% of the tuber’s fresh weight. The interconversion between starch and sugar affect potato quality, particularly the frying quality [4,5,6]. Light chips are a basic requirement for the potato processing industry. However, the consistent production of light chips throughout the year remains a major challenge [7]. Processing potatoes directly from cold storage (2–4 °C) into chips can reduce disease losses, extend marketability, and eliminate the need for dormancy-prolonging chemicals. Unfortunately, at low temperature, potato tubers are susceptible to cold-induced sweetening [8]. This phenomenon is an undesirable physiological process in which the rate of conversion of starch to reducing sugars (such as glucose and fructose) is accelerated in potato tubers [9]. As precursors of the Maillard reaction, a high concentration of reducing sugars such as glucose and fructose in potato tubers negatively affects frying quality. During frying, reducing sugars react with free amino acids (mainly asparagine), resulting in dark-colored, bitter-tasting substances. However, the more dangerous product of the Maillard reaction is acrylamide, a neurotoxin and suspected carcinogen [10,11]. Therefore, to reduce acrylamide content during frying, one effective way is to limit the accumulation of reducing sugars [12].

Starch is stored in almost all plant tissues, including leaves, roots, stems, flowers, and tubers. Extensive studies have shown that starch metabolism involves a series of enzymes, among which, amylases play an essential role in starch degradation [13,14,15]. The products of starch degradation provide essential support for plant growth and development and confer increased stress tolerance [16,17]. Starch degradation involves α-amylase (AMY) and β-amylase (BAM) [18]. AMY, as an endonucleoside hydrolase, hydrolyzes the α-1, 4-glycosidic bonds of polysaccharides (including starch) to generate oligosaccharides such as dextrin, maltotriose, maltose, and glucose [19,20,21]. Structurally, AMY possesses a (β/α)_8_-barrel catalytic domain, composed of three major domains [22,23,24]. The *AMY* family contains three subfamilies specific to the endosperm (subfamily I), cytoplasm (subfamily II), and chloroplast (subfamily III). Members of endosperm-specific subfamily I typically contain signal peptides which target the protein secretion pathway. In contrast, the functions of cytoplasm-specific subfamily II members are poorly characterized. Members of subfamily III typically contain a 400–500 amino acid (aa) expansion region and are predicted to act as chloroplast transport peptides. Research suggests that members of subfamily III are involved in the degradation of starch in leaves [25,26,27]. The transcription and enzymatic activities of *AMY* are regulated by a complex network that involves light, phytohormones, and stress factors [28,29]. Notably, *AMY* genes are controlled by gibberellin via gibberellin-induced MYBGA [30]. Furthermore, reports indicate that salt-stress-induced gibberellin-stimulated transcript (*OsGASR1*) regulated *AMY* gene expression [31]. Additionally, the *AMY3* gene participates in stress-induced starch degradation via abscisic acid, particularly through the AREB/ABF-SnRK2 kinase signaling pathway [32]. 

The research on potato starch and sugar metabolism is relatively comprehensive, particularly concerning metabolic enzymes [33,34,35]. Genetic engineering can be utilized to regulate starch and sugar metabolism by targeting these enzymes for inhibition or overexpression [36,37]. Starch degradation plays a pivotal role in the cold-induced sweetening process of potatoes. Within the potato genome, 77 loci have been identified to encode enzymes involved in starch metabolism [38]. Research suggests that AMY activity increases concomitantly with the accumulation of reducing sugars in potato tubers during the initial weeks of low-temperature storage [39]. Furthermore, the manipulation of *StAMY23* expression alters amylase activity and reducing sugar content in stored potato tubers [40,41]. The interaction between *StAmy23* and the amylase inhibitor gene (*SbAI*) regulates cold-induced sweetening in potato tubers by modulating amylase activity [42]. Improving potato tuber starch dynamics can be achieved through cloning and functionally characterizing genes related to starch synthesis and degradation, analyzing the promoter sequence regulatory elements of key starch metabolism genes, and elucidating the regulatory mechanism controlling various aspects of starch metabolism. 

Despite its importance, the *AMY* gene family has not been fully characterized in potato. In this study, we identified 20 *StAMY* genes across the potato genome. We analyzed the sequences, protein physicochemical properties, subcellular localizations, gene structures, conserved motifs, and phylogenetic evolution of each of the *StAMY* family genes. In addition, we investigated the functions and evolutionary characteristics of the *StAMY* gene family through promoter *cis*-acting element analysis and expression profiling. These results help to elucidate key genes involved in starch metabolism in potato tubers, lay the foundation for further research on *StAMY* genes, and provide a reference for starch improvement engineering in potato.

## 2. Materials and Methods

### 2.1. Identification of StAMY Genes

The whole-genome sequence and GFF3 annotation file were obtained from the Spud Database (http://spuddb.uga.edu/, accessed on 22 April 2024). Members of the *StAMY* gene family were identified via BLAST search using *Arabidopsis thaliana* AMY protein sequences as queries. To avoid missing any putative *StAMY* members, the potato protein sequences were queried using the Hidden Markov Model (HMM) file obtained from the Pfam database (http://pfam-legacy.xfam.org/, accessed on 22 April 2024), with default parameters. Following integration and redundancy elimination, the remaining sequences were used for further analyses. The chromosomal location map was created using TBtools. Coding sequence (CDS) length, isoelectric point (PI), and molecular weight (MW) were predicted using the Expasy website (https://www.expasy.org/resources/protparam, accessed on 22 April 2024). Protein subcellular localization was predicted using the WoLF PSORT Protein Subcellular Localization Prediction tool (https://wolfpsort.hgc.jp/, accessed on 22 April 2024).

### 2.2. Multiple Sequence Alignment and Phylogenetic Analysis of StAMY Genes

The *A. thaliana AMY* family genes were obtained from the NCBI database (https://www.ncbi.nlm.nih.gov/, accessed on 22 April 2024). All sequences of *AtAMY* and *StAMY* family genes were aligned, and a phylogenetic tree was constructed using the maximum likelihood (ML) method with default parameters in MEGA11.

### 2.3. Gene Structure and Conserved Motif Analysis of StAMY Genes

The structures and conserved domains of the *StAMY* genes were analyzed using Batch search (https://www.ncbi.nlm.nih.gov/Structure/cdd/wrpsb.cgi, accessed on 22 April 2024). Conserved motifs were analyzed using the MEME online tool (https://meme-suite.org/meme/, accessed on 22 April 2024), with the following parameters: the number of repetitions was set to zero or one and the maximum number of motifs was set to 10. Gene structures and conserved motifs were visualized using TBtools.

### 2.4. Promoter cis-Element Analysis of StAMY Genes

TBtools was used to extract the 2000 bp upstream sequences of *StAMY* genes. *Cis*-acting elements in the promoters of *StAMY* genes were predicted using the PlantCARE database (https://bioinformatics.psb.ugent.be/webtools/plantcare/html/, accessed on 22 April 2024). Finally, TBtools was used for visualization following statistical scanning.

### 2.5. Expression Analysis of StAMY Genes

The expression analysis of *StAMY* genes was performed using data obtained from the Spud Database (http://spuddb.uga.edu/, accessed on 22 April 2024). TBtools was used to construct heatmaps using log_2_-normalized fragments per kilo base of transcript per million mapped fragments (FPKM) values. The expression datasets included data from *S. tuberosum*-group Tuberosum RH89-039-16 (RH; mature tuber, young tuber, stolon, root, stem, leaf, flower) and *S. tuberosum-*group Phureja DM1-3 516 R44 (DM; heat-treated whole plant, salt-treated whole plant, mannitol-treated whole plant, β-aminobutyric acid [BABA]-treated leaves, *P. infestans*-treated leaves, benzothiadiazole [BTH]-treated leaves, abscisic acid [ABA]-treated whole plant, indole-3-acetic acid [IAA]-treated whole plant, gibberellic acid [GA3]-treated whole plant, 6-benzylaminopurine [BAP]-treated whole plant).

## 3. Results

### 3.1. Genome-Wide Identification of StAMY Genes

**Figure 1:**
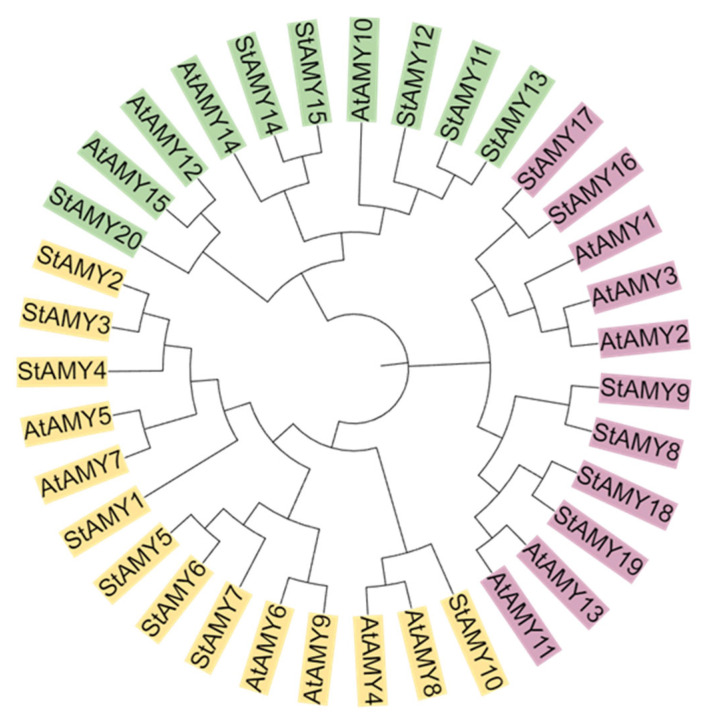
Phylogenetic tree of AMY protein sequences from *A. thaliana* (AtAMY) and *S. tuberosum* (StAMY).

A total of 20 *StAMY* genes were identified across the potato genome. The gene IDs, CDS lengths, start and end positions, protein lengths, MWs, and PIs of the *AMY* genes can be found in Table 1. The 20 StAMY proteins ranged in length from 373 aa to 1332 aa and ranged in weight from 41,687.07 Da to 150,181.53 Da. Their predicted theoretical PIs ranged from 4.9 to 6.89. The *StAMY* genes were found to be localized variously to the cytosol, vacuole, nucleus, chloroplast, mitochondrion, and peroxisome. Phylogenetic analysis of 20 StAMY and 15 AtAMY proteins revealed the presence of three distinct clades. As shown in Figure 1, the yellow clade included 14 members, including 8 StAMY and 6 AtAMY proteins; the pink clade included 11 members, including 6 StAMY and 5 AtAMY proteins; and the green clade included 10 members, including 6 StAMY and 4 AtAMY proteins. 

### 3.2. Chromosomal Locations of StAMY Genes

**Figure 2:**
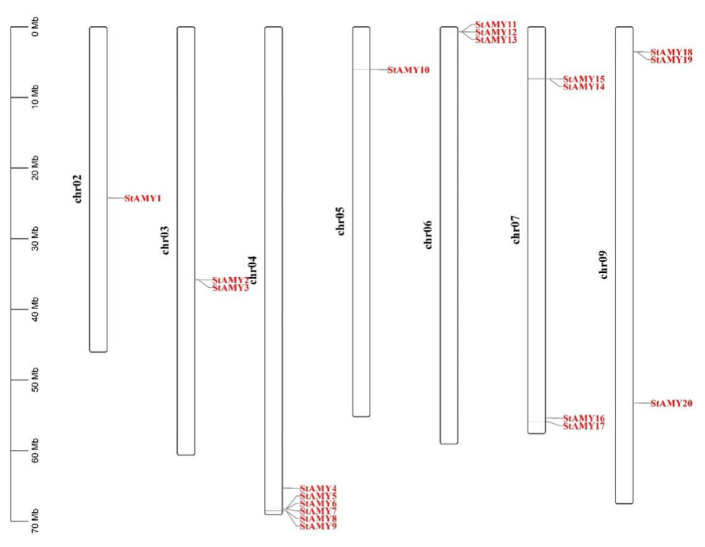
Chromosome location mapping of *StAMY* gene family in potato.

The 20 *StAMY* genes were found to be variously located on Chr02, Chr03, Chr04, Chr05, Chr06, Chr07, and Chr09 (Figure 2). Specifically, *StAMY1* was mapped to Chr02; *StAMY2* and *StAMY3* were mapped to Chr03; *StAMY4*, *StAMY5*, *StAMY6*, *StAMY7*, *StAMY8*, and *StAMY9* were mapped to Chr04 (which contained the greatest number of *StAMY* genes); *StAMY10* was mapped to Chr05; *StAMY11*, *StAMY12*, and *StAMY13* were mapped to Chr06; *StAMY14*, *StAMY15*, *StAMY16*, and *StAMY17* were mapped to Chr07; and *StAMY18*, *StAMY19*, and *StAMY20* were mapped to Chr09.

### 3.3. Gene Structures and Conserved Motifs of StAMY Genes

Exon–intron analysis can help researchers to understand the structural evolution of gene families. Here, we studied the exon–intron structures of *StAMY* genes using both genomic and coding DNA sequences. Overall, the number, length, and distribution of exons and introns differed among genes. *StAMY2*, *StAMY3*, *StAMY5*, *StAMY6*, *StAMY7*, *StAMY8*, *StAMY9*, *StAMY11*, *StAMY12*, *StAMY13*, *StAMY14*, *StAMY15*, *StAMY16*, *StAMY17*, *StAMY*18, and *StAMY*19 exhibited similar sequence lengths but different numbers of exons. 

**Figure 3:**
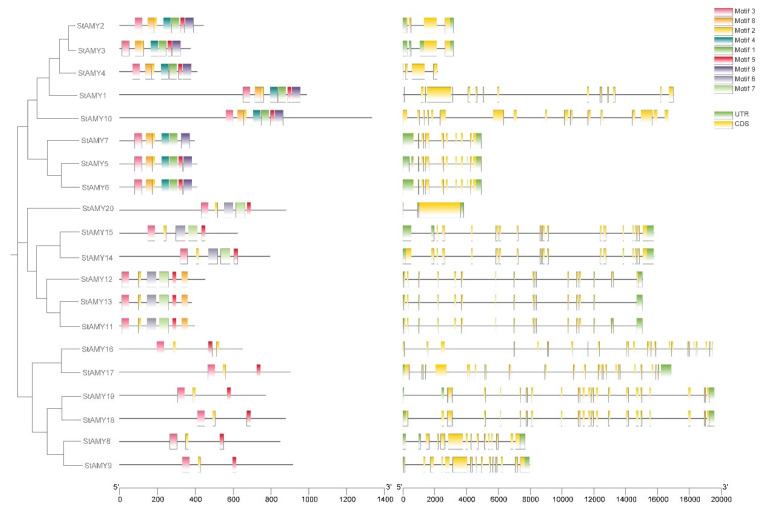
Analysis of gene structures and motifs of *StAMY* gene family members in potato. CDS, coding sequences. UTR, untranslated region.

In addition, StAMY family protein architecture was investigated using amino acid sequences. According to MEME motif analysis, the common motifs tended to cluster into the same groups, and many of the motifs were clade- and group-specific (Figure 3). Group I (*StAMY1*, *StAMY*2, *StAMY*3, *StAMY*4, *StAMY*5, *StAMY*6, *StAMY*7, and *StAMY10*) contained seven of the same motifs, including motifs 1, 2, 3, 4, 5, 8 and 9. Group II (*StAMY*11, *StAMY*12, *StAMY*13, *StAMY*14, *StAMY*15, and *StAMY20*) contained motifs 2, 3, 5, 6, 7, and 8. Group III (*StAMY8*, *StAMY9*, *StAMY16*, *StAMY17*, *StAMY18*, and *StAMY19*) contained motifs 2, 3, and 5.

### 3.4. Promoter cis-Acting Elements of StAMY Genes

**Figure 4:**
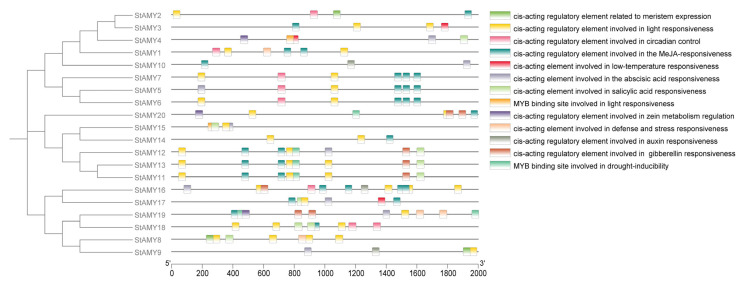
Analysis of *cis*-acting elements in the promoters of *StAMY* genes in potato.

Analyzing *cis*-acting elements present in the promoters of genes can help researchers to understand both gene function and regulation. A total of 13 *cis*-acting elements were identified across *StAMY* gene family promoters, including those associated with plant growth and development, phytohormone signaling, defense, and stress response. Specifically, the promoters of *StAMY* genes were found to contain 70 phytohormone-responsive *cis*-elements (including those responsive to ABA, methyl jasmonate [MeJA], salicylic acid [SA], auxin [Aux], and GA3), 44 light-responsive *cis*-elements, 3 low-temperature-responsive *cis*-elements, 6 drought-responsive MYB binding sites, 4 defense- and stress-responsive *cis*-elements, 8 circadian control *cis*-elements, 3 zein metabolism-regulating *cis*-elements, and 3 meristem-specific *cis*-elements. The most common elements were those related to the response to light and MeJA (Figure 4).

### 3.5. Tissue-Specific Expression Profiles of StAMY Genes

**Figure 5:**
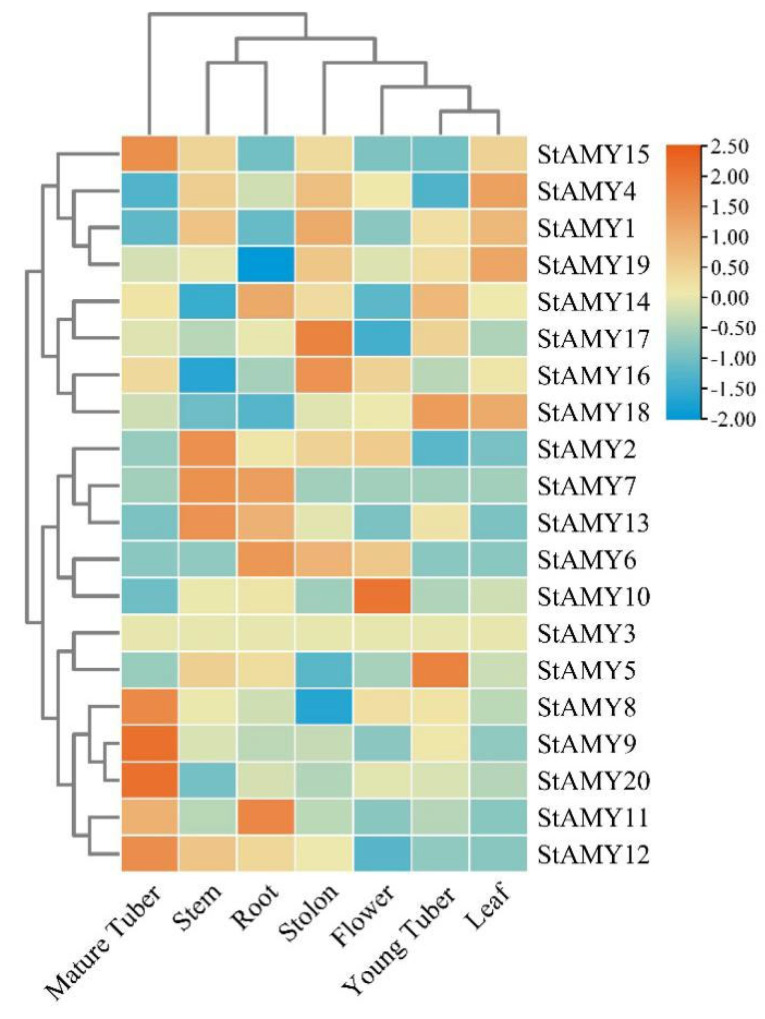
Tissue-specific expression patterns of *StAMY* genes.

To study the tissue-specific expression of *StAMY* genes, we analyzed publicly available RNA-seq data obtained from the Spud Database (https://spuddb.uga.edu/, accessed on 22 April 2024). In general, the various *StAMY* genes exhibited tissue-specific expression (Figure 5). Specifically, *StAMY8*, *StAMY9*, *StAMY12*, *StAMY15*, and *StAMY20* were highly expressed in mature tubers; *StAMY2*, *StAMY7*, and *StAMY13* were highly expressed in stems; *StAMY6* and *StAMY11* were highly expressed in roots; *StAMY16* and *StAMY17* were highly expressed in stolons; *StAMY10* was highly expressed only in flowers; *StAMY5* and *StAMY18* were highly expressed in young tubers; and *StAMY4*, *StAMY18*, and *StAMY19* were highly expressed in leaves. Notably, *StAMY*3 was not found to be expressed in any of the tested tissues.

### 3.6. Changes in StAMY Gene Expression in Response to Biotic and Abiotic Stress

**Figure 6:**
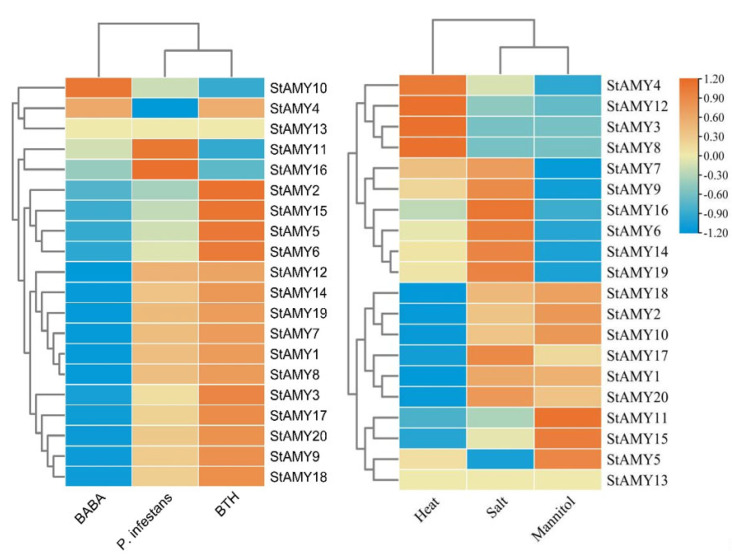
Changes in the expression patterns of *StAMY* genes in response to biotic and abiotic stress.

Changes in *StAMY* gene expression were evaluated in whole plants or leaves exposed to BABA, BTH, *P. infestans*, heat, salt, and mannitol (Figure 6). *StAMY10* exhibited upregulated expression in response to BABA treatment, whereas *StAMY1, StAMY3, StAMY7*, *StAMY8*, *StAMY9*, *StAMY12*, *StAMY14*, *StAMY17*, *StAMY18*, *StAMY19*, and *StAMY20* were downregulated. *StAMY11* and *StAMY16* exhibited upregulated expression in response to *P. infestans* infection, whereas the expression of *StAMY4* was downregulated. *StAMY2*, *StAMY5*, *StAMY6*, and *StAMY15* exhibited upregulated expression in response to BTH treatment. *StAMY3*, *StAMY4*, *StAMY8*, and *StAMY12* exhibited upregulated expression in response to heat treatment, whereas *StAMY1*, *StAMY2*, *StAMY10*, *StAMY18,* and *StAMY20* were downregulated. *StAMY6*, *StAMY14*, *StAMY16*, and *StAMY19* exhibited upregulated expression in response to salt treatment. *StAMY5*, *StAMY11*, and *StAMY15* exhibited upregulated expression in response to mannitol treatment, whereas *StAMY6*, *StAMY7*, *StAMY9*, *StAMY14*, and *StAMY19* were downregulated. 

### 3.7. Changes in StAMY Gene Expression in Response to Phytohormones

**Figure 7:**
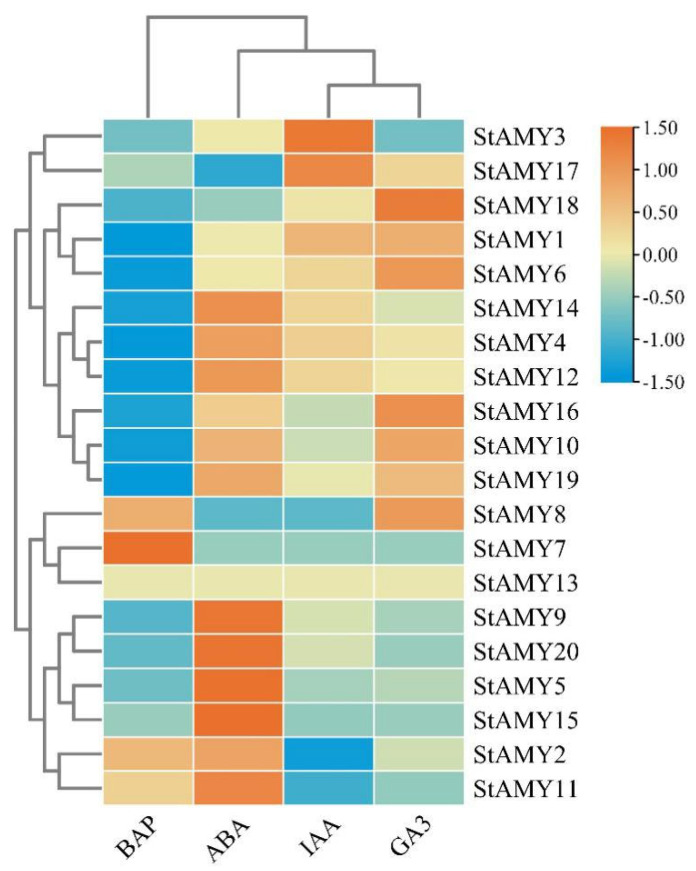
Changes in the expression patterns of *StAMY* genes in response to 6-benzylaminopurine (BAP), abscisic acid (ABA), indole-3-acetic acid (IAA), and gibberellic acid (GA3).

Changes in *StAMY* gene expression were evaluated in whole plants exposed to BAP, ABA, IAA, and GA3 (Figure 7). *StAMY7* exhibited upregulated expression in response to BAP treatment, whereas *StAMY1*, *StAMY4*, *StAMY6*, *StAMY10*, *StAMY12*, *StAMY14*, *StAMY16*, and *StAMY19* were downregulated. *StAMY5*, *StAMY9*, *StAMY15*, and *StAMY20* exhibited upregulated expression in response to ABA treatment, whereas the expression of *StAMY17* was downregulated. *StAMY3* and *StAMY17* exhibited upregulated expression in response to IAA treatment, whereas *StAMY2* and *StAMY11* were downregulated. *StAMY18* exhibited upregulated expression in response to GA3 treatment.

## 4. Discussion

In order to minimize processing losses caused by cold-induced sweetening during the low-temperature storage of potato tubers, preventing the conversion of starch into reducing sugars plays a crucial role in improving the processing quality of potato chips [43]. Since α-amylase (*AMY*) is the primary starch-degrading enzyme in potato, it is critically important to identify, functionally characterize, and study its role in potato tuber quality. In this study, we used whole-genome sequencing data to identify members of the *StAMY* gene family. In addition, we evaluated their structures and functions, providing a theoretical basis for further research on the role of the *AMY* gene family in potato growth and development.

Previous studies have found that many plants, including barley, quinoa, apple, and others, contain multigene *AMY* families [44,45,46]. Here, we identified 20 *AMY* genes in the potato genome, which were unevenly distributed on the seven potato chromosomes (Figure 2). In addition, the StAMY proteins exhibited varied conserved motif compositions, likely due to functional differentiation of the *StAMY* family during evolution. Alternative splicing can result in a polymorphic protein structure, as well as functional and transcriptional differentiation [47]. High sequence similarity and close evolutionary relationships were observed between *StAMY2* and *StAMY3*, *StAMY5*, *StAMY6*, and *StAMY7*; *StAMY8* and *StAMY9*, *StAMY11*, *StAMY12*, and *StAMY13*; and *StAMY14* and *StAMY15*, *StAMY18,* and *StAMY19*. However, gene structure analysis indicated that many of these genes exhibited differences in intron–exon structure, resulting in different functions and expression patterns (Figure 3). The phylogenetic tree revealed that the protein structures of the three branches were relatively similar between *A. thaliana* and potato, suggesting that members of each branch may have originated from a common ancestor (Figure 1).

Analysis of promoter *cis*-elements can help determine the function of specific genes. Here, we identified a number of defense-, stress-, and phytohormone-responsive *cis*-elements in the promoters of *StAMY* genes (Figure 4), suggesting that these genes may be involved in the plant response to stress, as well as hormonal and developmental signaling. We investigated the expression profiles of 20 *StAMY* genes under biotic and abiotic stress using DM data obtained from the Spud Database (http://spuddb.uga.edu/, accessed on 22 April 2024). Notably, *StAMY3*, *StAMY4*, *StAMY8*, and *StAMY12* exhibited upregulation in response to heat stress, suggesting that these genes play a positive role in the heat stress response. *StAMY6*, *StAMY14*, *StAMY16*, *StAMY17*, *StAMY19*, and *StAMY20* exhibited upregulated expression in response to salt stress, suggesting that these genes similarly play a positive role in the salt stress response. In addition, *StAMY5*, *StAMY11* and *StAMY15* were significantly upregulated in response to mannitol treatment, suggesting that these genes play an active role in the drought stress response.

BTH is a synthetic chemical analog of SA and is a potent inducer of plant defenses. BTH has been used to induce protection against diseases in various crops, including potato, tomato, rice, and wheat [48]. We found that *StAMY2*, *StAMY5*, *StAMY6*, and *StAMY15* exhibited significantly upregulated expression in response to BTH treatment. BABA is a phytochemical inducer which can induce plant defense responses to an array of biotic and abiotic stressors [49]. We found *StAMY10* exhibited significant upregulation in response to BABA, whereas *StAMY1*, *StAMY3*, *StAMY7*, *StAMY8*, *StAMY9*, *StAMY12*, *StAMY14*, *StAMY17*, *StAMY18*, *StAMY19*, and *StAMY20* were downregulated. These results suggest that these genes may play a role in plant defense and stress resistance. *P. infestans* is one of the most serious pathogens of potatoes, tomatoes, and other solanaceous crops [50,51]. We observed that *StAMY11* and *StAMY16* exhibited upregulated expression in response to *P. infestans* infection, indicating that these genes are likely involved in the pathogen defense response.

Phytohormones regulate plant adaptation and defense through a series of signal transduction processes [52]. To investigate the effect of phytohormone signaling on *StAMY* gene expression, *StAMY* gene expression was examined in ABA-, IAA-, GA3-, and BAP-treated potato plants. Among these, GA3 and ABA are the most relevant to tuber formation, with GA3 inhibiting and ABA promoting tuber formation [53]. We found that the expression of *StAMY5*, *StAMY9*, *StAMY11*, *StAMY15*, and *StAMY20* was upregulated in response to ABA treatment. In addition, GA3 treatment upregulated the expression of *StAMY6*, *StAMY8*, *StAMY16*, and *StAMY18*. The promoters of *StAMY* genes were found to contain a variety of phytohormone-responsive *cis*-acting elements, including those responsive to GA3 and ABA. The expression of *StAMY3* and *StAMY17* was upregulated in response to IAA treatment. Notably, *StAMY17* was found to be highly expressed in stolons, suggesting that this gene may promote potato tuber growth. However, this hypothesis requires experimental verification. BAP was the first synthetic cytokinin [54]. We observed that the expression of *StAMY7* was upregulated in response to BAP treatment, whereas *StAMY1*, *StAMY4*, *StAMY6*, *StAMY10*, *StAMY12*, *StAMY14*, *StAMY16*, and *StAMY19* were downregulated. Taken together, these results suggest that many of the *StAMY* genes are likely involved in the phytohormone-mediated growth and development of potatoes.

In summary, we identified 20 *StAMY* genes and analyzed their phylogenetic relationships, gene structures, functional motifs, and expression patterns. *StAMY8*, *StAMY9*, *StAMY12*, and *StAMY20* were specifically expressed in mature tubers, suggesting that they may be involved in starch and sugar metabolism. Different *StAMY* gene family members were significantly upregulated or downregulated in response to a variety of stressors and exogenous phytohormones. Additional studies should be conducted to further investigate the roles of *StAMY* genes in the regulation of starch metabolism, growth, and development. 

## Figures and Tables

**Table 1:** Molecular characteristics of identified *StAMY* genes in potato.

Gene ID	CDS Length (bp)	Start–End Positions	Protein Length (aa)	Molecular Weight (Da)	Isoelectric Point (PI)	Subcellular Localization
*StAMY1*	2964	24230843-24247840	987	112,118.25	5.77	cytosol
*StAMY2*	1326	35837209-35840386	441	49,246.7	5.43	vacuole
*StAMY3*	1122	35837209-35840386	373	41,687.07	5.17	cytosol
*StAMY4*	1224	65328724-65330894	407	45,723.43	5.83	chloroplast
*StAMY5*	1224	68257647-68262571	407	46,346.11	6.74	cytosol
*StAMY6*	1224	68257647-68262571	407	46,346.11	6.74	cytosol
*StAMY7*	1188	68257647-68262571	395	44,870.42	6.67	cytosol
*StAMY8*	2547	68516172-68508497	848	96,766.73	4.9	nucleus
*StAMY9*	2745	68516457-68508497	914	104,165.09	5.09	chloroplast
*StAMY10*	3999	6053915-6037246	1332	150,181.53	6.05	mitochondrion
*StAMY11*	1185	715327-700273	394	45,248.83	6.27	peroxisome
*StAMY12*	1350	715327-700273	449	51,028.05	6.1	peroxisome
*StAMY13*	1143	715327-700273	380	43,513.8	6.27	peroxisome
*StAMY14*	2382	7370232-7354469	793	89,389.69	5.54	nucleus
*StAMY15*	1866	7370232-7354469	621	70,484.13	5.19	nucleus
*StAMY16*	1947	55405743-55386292	648	73,772.35	6.89	chloroplast
*StAMY17*	2709	55936361-55953226	902	103,776.35	6.4	cytosol
*StAMY18*	2631	3555467-3535898	876	100,067.77	5.02	chloroplast
*StAMY19*	2316	3555467-3535898	771	88,867.57	4.94	cytosol
*StAMY20*	2637	53271759-53267937	878	97,998.1	6.8	chloroplast

## Data Availability

Data are contained within the article.

## Abbreviations

α-amylase*AMY*; β-aminobutyric acid, BABA; benzothiadiazole, BTH; *Phytophthora infestans*, *P. infestans*; abscisic acid, ABA; indole-3-acetic acid, IAA; gibberellic acid, GA3; 6-benzylaminopurine, BAP.
